# Integrating Cover Crops and Manure to Boost Goji Berry Yield: Responses of Soil Physicochemical Properties and Microbial Communities

**DOI:** 10.3390/microorganisms13030696

**Published:** 2025-03-20

**Authors:** Haonan Chen, Fang Wang, Yamiao Gao, Yaran Ma, Lizhen Zhu, Xiongxiong Nan

**Affiliations:** 1 College of Geographical Sciences and Planning, Ningxia University, Yinchuan 750021, China; chn1504924790@163.com (H.C.); gaoym11@stu.nxu.edu.cn (Y.G.); myyr@stu.nxu.edu.cn (Y.M.); 2 China-Arab Joint International Research Laboratory for Featured Resources and Environmental Governance in Arid Regions, Yinchuan 750021, China; 3 State Key Laboratory of Efficient Production of Forest Resources, Yinchuan 750002, China; lizhenzhu916@163.com

**Keywords:** *Lycium barbarum* L., forage radish, organic manure, microbial diversity, network complexity, structural equation modeling

## Abstract

A sustainable Goji berry (*Lycium barbarum* L.) planting system that integrates forage radish cover crops (*Raphanus sativus* L.) and animal manure has been established in northwestern China. This study investigated the effects of different cropping systems and manure application levels on soil physicochemical properties, microbial community structure, and *L. barbarum* yield under field conditions. A split-plot design was used, with the main-plot treatments consisting of two cropping systems and the sub-plot treatments involving three manure application levels. The results showed that compared to *L. barbarum* monocropping, cover cropping with *R. sativus* led to a decrease in soil bulk density (1.90%) and increase in soil electrical conductivity (11.5%), nutrient contents (total N and available N, P, and K: 30.3–138%), and microbial biomass (C: 79.0%; N: 184%). Cover cropping additionally enhanced the community diversity and richness of soil bacteria. Beta-diversity analysis revealed significant differences in bacterial rather than fungal community composition among various treatments. The bacterial network showed a lower ratio of positive to negative correlations and reduced complexity in response to cover cropping, which contrasted with fungal network patterns. Integration of cover cropping and medium manure application increased fruit yield by 8.71%. Cover crops and manure influenced soil microbial diversity mainly through their positive effects on soil total and available N contents.

## 1. Introduction

Soil microbes play an active role in soil health maintenance and can serve as promising indicators of soil fertility [1,2]. Myriad microbes drive soil biogeochemical processes, such as nutrient cycling and organic matter decomposition, leading to changes in the soil environment. In agroecosystems, rational management practices can boost sustainable crop production by reshaping the soil microbiota [3,4]. Therefore, it is crucial to understand how agricultural management practices affect soil microbial abundance, community structure, and species interactions. Unveiling soil microbial responses to agricultural management practices can provide clues to soil fertility changes and foster sustainable use of land resources, promoting the development of regional agriculture.

Goji berry (*Lycium barbarum* L.) is a perennial shrub of the family Solanaceae, native to Ningxia, China [5]. This functional crop is favored by consumers worldwide because of its food and medicinal values. As of 2021, the planting area and dried fruit yield of *L. barbarum* in Ningxia accounted for 46% and >50% of the national total, respectively [6]. While the market demand for *L. barbarum* is growing, soil degradation in old orchards has led to fruit yield loss and quality decline. This problem has arisen mainly due to the long-term implementation of intensive management practices, such as clear tillage, flood irrigation, and excessive chemical fertilization with limited organic amendment [7]. Environmentally friendly strategies should be adopted to sustain high yield of *L. barbarum*.

Integrating cover crops into orchard systems is an advanced soil management method and a sustainable agricultural development model aimed at improving orchard quality and production efficiency. Compared to monocropping, the use of appropriate cover crops can ameliorate the soil environment by increasing water infiltration, preventing soil erosion, and enhancing microbial activity [8,9]. Cover crops also promote the soil nitrogen (N) cycle and carbon (C) storage, conveying benefits to fruit quality and yield [10]. In particular, cover crops affect the dynamics of soil microbiota in orchards as a consequence of soil environmental changes [11]. For example, cover cropping with ryegrass (*Lolium perenne*) and white clover (*Trifolium repens*) led to increased soil bacterial and fungal diversity in apple orchards [12]. Higher bacterial richness and diversity occurred in kiwi orchards planted with *L. perenne* and *T. repens* [13]. In contrast, the diversity and abundance of soil bacteria in persimmon orchards decreased in response to cover cropping with round leaf cassia (*Chamaecrista rotundifolia*) and perennial peanut (*Arachis pintoi*) [14]. These findings highlight distinct responses of soil microbiota to various cover crops. Additionally, animal manure is often applied as an organic soil amendment in cover crop systems to promote soil improvement and crop growth [15]. How this integrated practice affects fruit production remains largely unexplored.

Forage radish (*Raphanus sativus* L.) is a cruciferous annual plant widely used as cover crop in rice fields and tea gardens in southern China [16,17]. *R. sativus* shows compatibility with *L. barbarum* in terms of their growth habits and nutrient requirements. In recent years, a sustainable planting system that integrates *L. barbarum* (economic crop) and *R. sativus* (cover crop) has been established in the arid regions of northwestern China [5]. A major question arising from this significant shift in cropping system is the response patterns of soil microbial communities. Previous research has evaluated the influence of monocropping on the fruit yield, aboveground biomass, and nutrient or water use efficiency of *L. barbarum* [18]. However, microbially mediated mechanisms underlying the changes of fruit yield in the *L. barbarum*/*R. sativus* planting system remain unclear.

Here, we ascertained the long-term effects of cover cropping and manure application on soil physicochemical properties, microbial community structure, and fruit yield in an *L. barbarum* orchard. We hypothesized that the following: (1) Integrated cover cropping and manure application would increase soil nutrient supply, creating a favorable growth environment for *L. barbarum*, and (2) soil microbial biomass, diversity, and network complexity would positively respond to cover cropping and manure application, leading to improved fruit yield.

## 2. Materials and Methods

### 2.1. Field Site and Experimental Design

**Figure 1:**
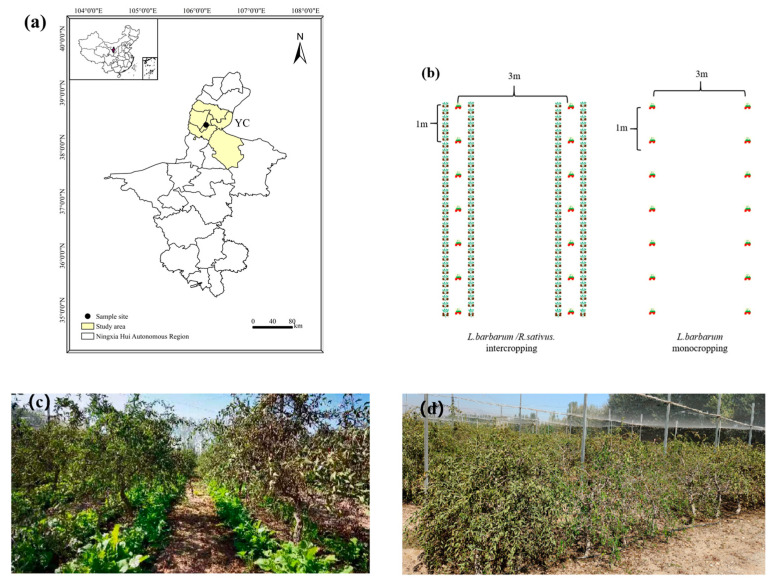
Experimental site and cropping systems. (**a**) Location of the experimental site in Yinchuan (YC), (**b**) schemes of two cropping systems, (**c**) cover cropping with *R. sativus*, and (**d**) monocropping of *L. barbarum*.

The field experiment was started in 2016 at the demonstration base of Wolfberry Engineering Technology Research Center, State Forestry Administration (Yinchuan, Ningxia, China; Figure 1a). The experimental site (38°24′ N, 106°10′ E) is located in the mid-temperate zone with a continental climate. The winter and spring are dry, with high winds in all four seasons. The temperature shows large diurnal variations with an annual mean of 8.5 °C. The mean annual rainfall and evaporation are 200 and 1883 mm, respectively, and the mean annual frost-free period ranges between 160–170 days. The major soil type is classified as Arenosols (Harmonized World Soil Database, version 2.0).

The varieties of *L. barbarum* and *R. sativus* used in the study were ‘Ningqi No. 1’ and ‘Longipinnatus’, respectively. In the conventional monocropping system, *L. barbarum* was planted at a spacing of 1 m between plants and 3 m between rows; the inter-row spaces were left fallow with no tillage applied, except for routine weeding and maintenance. In the cover cropping system, *R. sativus* was sown along the *L. barbarum* rows at the beginning of autumn, that is, the late growth stage of *L. barbarum* (summer dormancy period). The spacing of *R. sativus* plants was 20 cm, with a 20-cm distance from the *L. barbarum* rows. The sowing depth of *R. sativus* was 1–2 cm below the soil surface, and the sowing density ranged from 33,300 to 37,500 plants·ha^−1^. All *R. sativus* plants were retained in the field, where they would succumb to frost damage and die by late December, decomposing in situ during the following spring. All other field management practices remained consistent with those of the conventional cropping system.

This experiment used a split-plot design with two treatment factors. The primary treatment factor was the cropping system: conventional monocropping of *L. barbarum* (M) and integrated cover cropping with *R. sativus* (I). The secondary treatment factor was the manure application rate based on the amount applied by local farmers: no manure application (M_0_), moderate manure application (2 kg per plant; M_1_), and high manure application (4 kg per plant; M_2_). A completely randomized block design was used, with each treatment replicated three times. The plot size was 120 m^2^ each (20 m long × 6 m wide). For chemical fertilization, urea, diammonium phosphate, and potassium sulfate were used. *L. barbarum* plants were top-dressed in the root zone from late April to late June and from late June to late July. Each plant received 120 g of N, 60 g of P_2_O_5_, and 75 g of K_2_O per application, with a total of two applications during the entire growth period. Decomposed sheep manure (0, 6660, and 13,320 kg·ha^−1^) was applied during the leaf expansion stage of *L. barbarum*, along a circular trench (30 cm deep × 30 cm wide) dug 30 cm away from the plant trunk.

### 2.2. Soil Sampling, Testing Methods, and Fruit Yield Estimation

Soil samples were collected from a depth of 0–20 cm in early August 2021. Within the *R. sativus* rows, five representative sampling points were selected from each plot. Soil samples from the same plot were thoroughly mixed, and impurities such as gravel and plant residues were removed. The soil samples were sieved through a 2 mm mesh and each sample was divided into four parts by quartering. A subsample was taken from each part and four subsamples were pooled together to give a composite sample, which was rapidly frozen in liquid nitrogen and stored in a −80 °C freezer for microbial community analysis. Another composite soil sample was used to determine soil properties.

Soil properties were analyzed using standard procedures [19]. Soil pH was measured by potentiometry and electrical conductivity (EC) was determined by conductometry (water/soil = 2.5:1, *v*/*w*). The Kjeldahl method were used for determination of total N (TN). Microbial biomass C (MBC) and N (MBN) were determined by the chloroform fumigation method. Available nitrogen (AN) was analyzed using a continuous flow analyzer (Model 5000; SKALAR, Breda, The Netherlands) after sample extraction with a 1 mol·L^−1^ KCl solution. Available phosphorus (AP) was determined by molybdenum–antimony anti-colorimetry with a 0.5 mol·L^−1^ NaHCO_3_ solution. Available potassium (AK) was quantified using flame photometry after extraction with 1 mol·L^−1^ NH_4_OAc solution. Bulk density (BD) was determined using the cutting ring method. The yield of *L. barbarum* fruits was measured in the period from the maturity of the first batch to the end of the fruiting season. Three plants were selected from each plot, and the cumulative yield of their fresh fruits was recorded.

### 2.3. DNA Extraction, PCR Amplification, and Sequencing

The extraction of total genomic DNA from soil samples was performed using the E.Z.N.A.^®^ Soil DNA Kit (Omega Biotek, Norcross, GA, USA) as per the manufacturer’s instructions. The V3–V4 variable regions of the 16S bacterial ribosomal RNA genes were amplified by PCR with the primer set 338F/806R (forward: 5′-ACTCCTACGGGAGGCAGCA-3′ and reverse: 5′-GGACTACHVGGGTWTCTAAT-3′) [20]. PCR amplification of the internal transcribed spacer (ITS) region in the fungal nuclear ribosomal RNA genes was performed with the primer set ITS1F/ITS2R (forward: 5′-CTTGGTCATTTAGGGAAGTAA-3′ and reverse: 5′-GCTGCGTCTTCATCGATGC-3′) [21].

PCR reactions for bacteria and fungi were prepared using TransStart FastPfu DNA Polymerase (TransGen Biotech, Beijing, China) and TaKaRa rTap Polymerase (TaKaRa, Dalian, China), respectively. The PCR conditions were as follows: 95 °C for 3 min, followed by 27–35 cycles of 95 °C for 30 s, 55 °C for 30 s, and 72 °C for 30 s, with a final extension step at 72 °C for 10 min. After a quality check, PCR amplicons were used for Illumina high-throughput sequencing at Majorbio (Shanghai, China) to generate 50 bp paired-end reads.

### 2.4. Data Processing and Analysis

The raw reads from high-throughput sequencing were processed on the Majorbio Cloud Platform (https://www.majorbio.com/). Quality control was carried out using fastp v0.20.0 (https://github.com/OpenGene/fastp) [22], and chimeras were removed using FLASH v1.2.7 (http://www.cbcb.umd.edu/software/flash) [23]. (1) A 50 bp window was set to filter bases of reads with a tail mass value < 20. If the average mass value in the window was <20, the back-end bases were trimmed from the window. Short reads below 50 bp were filtered, with N-bases containing reads removed. (2) After quality control, paired-end reads with a minimum overlap length of 10 bp were merged into a sequence. (3) The maximum mismatch ratio allowed in the overlap area of spliced sequence was 0.2, and non-conforming sequences were screened out. (4) Samples were differentiated by barcode and primers at both ends of the sequence, and sequence direction was adjusted. The number of allowed mismatches in a barcode was 0 and the maximum number of allowed mismatches in a primer was 2. UPARSE v7.1 (http://drive5.com/uparse/) was used for clustering sequences into operational taxonomic units (OTU) at a 97% similarity level [24,25]. The RDP classifier v2.2 (http://www.arb-silva.de/)was used to annotate taxonomy for each representative sequence of bacterial 16S rRNA and fungal ITS genes based on the Silva v138 and Unite8.0 databases, respectively, with a threshold of 70% [26].

Sample rarefaction curves were created using Rstudio v4.3.1 (https://www.r-project.org/) to determine whether sequencing depth could suffice to cover all microbial taxa in the samples. Four alpha-diversity indices (Ace, Chao1, Simpson, and Shannon) under random sampling were calculated using mothur v1.30.2 (https://mothur.org/wiki/calculators/, accessed on 15 November 2022.). The means of alpha-diversity indices in each treatment group were compared by two-way analysis of variance (ANOVA) followed by Duncan’s multiple range test at the 5% level, and the analysis was implemented in the Data Processing System v9.01 (http://www.chinadps.net/). Beta-diversity analysis was performed by principal co-ordinates analysis (PCoA) based on Bray–Curtis dissimilarity using normalized OTU data that were rarefied to the minimum number of read counts across samples. Permutational multivariate analysis of variance (PERMANOVA) was used to evaluate the effects of different cropping systems on microbial community composition. Bacterial and fungal community compositions were characterized based on the relative abundances of representative taxa at the phylum and genus levels. The means of taxa relative abundances in each treatment group were obtained and the taxa with a relative abundance < 1% were combined into “others”.

To reveal the interactions among microbial taxa, the top 400 genera of bacteria and fungi were selected based on their relative abundances. Spearman correlation coefficients between the selected bacterial or fungi genera were calculated to construct co-occurrence networks. The correlation coefficient matrix was created based on *p* < 0.05, |*ρ*| ≥ 0.5, and omission of node self-connections [27]. Gephi v0.9.2 (https://gephi.org/) was used to visualize the networks. The influence of agricultural management practices on soil properties, microbial diversity, and *L. barbarum* yield was disentangled by structural equation modeling performed using Amos v26.0 (IBM, Armonk, NY, USA).

## 3. Results

### 3.1. Changes in Soil Properties and Fruit Yield

Two-way ANOVA results showed that different cropping systems significantly affected soil BD, TN, AN, AP, and MBC (Table 1). Compared to *L. barbarum* monocropping, cover cropping with *R. sativus* led to distinct increase in soil TN, AN, AP, MBC, and MBN contents (by 30.3–184%), accompanied by decrease in soil BD (by 1.90%; *p*-values < 0.05). Manure application significantly affected soil EC and AN. The interaction effect between cropping system and manure application was significant on soil AN only. Under the same manure level, soil TN, MBC, and MBN contents increased by 112–261% in the IM_1_ treatment compared to the MM_1_ treatment. Soil EC, AP, and MBC contents increased by 22.8%, 201%, and 103%, respectively, in the IM_2_ treatment relative to the MM_2_ treatment.

**Figure 2:**
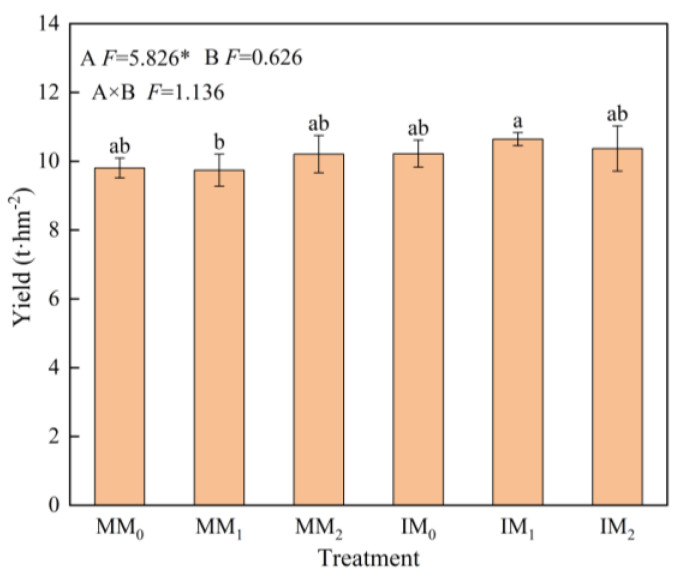
Fruit yield of *Lycium barbarum* under various treatments. Error bars represent the standard deviation of the mean (*n* = 3). Different letters above the error bars indicate significant difference among treatments (Duncan’s multiple range test: *p* < 0.05). MM_0_, MM_1_, and MM_2_ are the monocropping treatments and IM_0_, IM_1_, and IM_2_ are the cover cropping treatments, with zero, medium, and high levels of organic manure, respectively (* *p* < 0.05).

The yield of fresh *L*. *barbarum* fruits under cover cropping was 4.97% higher than that of the monocropping system (Figure 2). Under the same level of organic manure, the fruit yield of the IM_1_ treatment increased by 8.71% compared to that under the MM_1_ treatment (*p* < 0.05). Yield increase also occurred in other cover cropping treatments, albeit not significantly.

### 3.2. Soil Microbial Diversity Patterns

After deletion of low-quality reads, a total of 315,995 clean tags of bacteria were obtained from 18 soil samples, including 156,432 effective tags (>42% of clean tags; Table S1). For the fungi, the total number of clean tags was 420,398, including 220,972 effective tags (>37% of clean tags). For both bacterial and fungal communities, the number of observed OTUs (i.e., Sobs) in all soil samples increased rapidly with the increase in sequencing data volume. When the number of reads sampled reached 3500, the rarefaction curves of the soil samples basically leveled off (Figure S1). The results indicate that the sequencing depth was sufficient to capture the information of microbial species composition in the samples.

**Figure 3:**
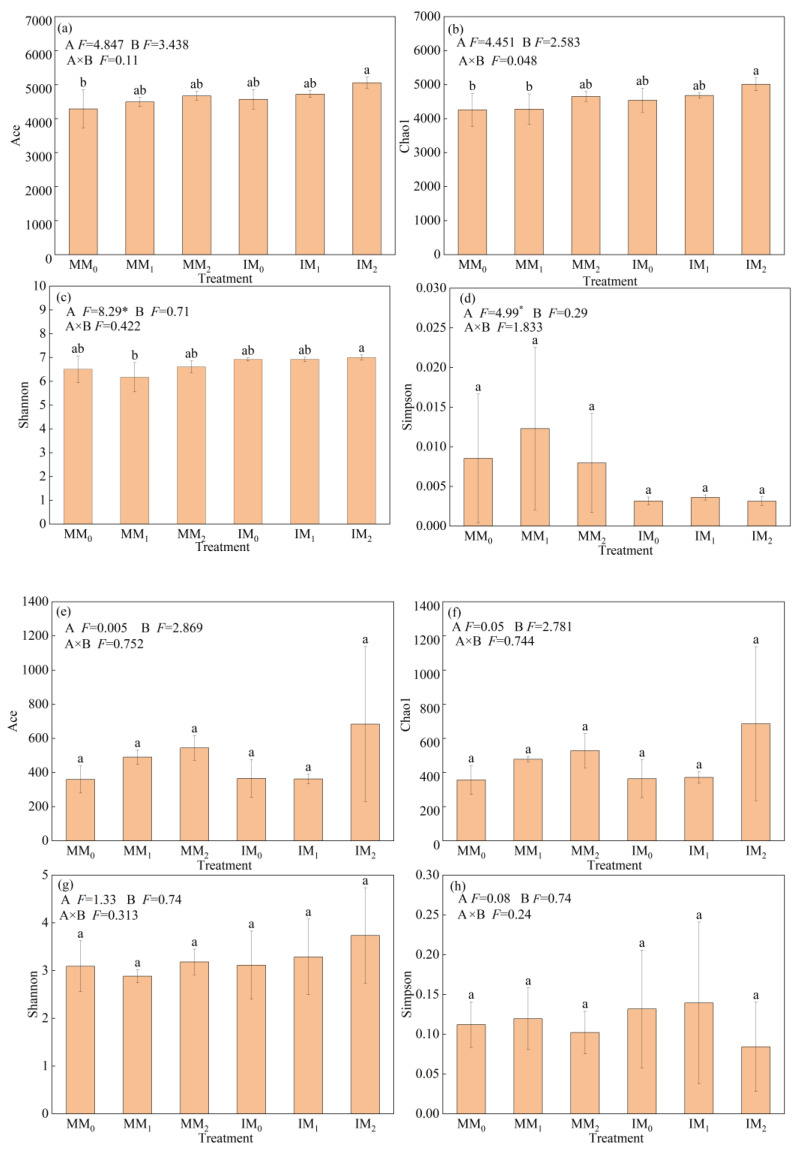
Alpha-diversity of (**a**–**d**) bacterial and (**e**–**h**) fungal communities in soil samples under various treatments. Error bars represent the standard deviation of the mean (*n* = 3). Different letters above the error bars indicate significant difference among treatments (Duncan’s multiple range test: *p* < 0.05) (* *p* < 0.05).

With respect to microbial alpha-diversity, Ace and Chao1 were used to measure species richness; the Shannon and Simpson indices were used to measure community diversity. The alpha-diversity indices of soil bacterial and fungal communities showed differential responses to various treatments (Figure 3). Compared to monocropping, the Ace, Chao1, and Shannon indices of bacterial communities increased by 6.32–6.57%, whereas the Simpson index decreased by 62.3% under cover cropping (Figure 3a–d). However, there were no significant differences in fungal alpha-diversity indices between the two cropping systems (Figure 3e–h). Under the same manure level, bacterial alpha-diversity indices were all higher in the IM_2_ treatment relative to the MM_2_ treatment, albeit not significantly. The Shannon index of bacterial communities was slightly higher in the IM_1_ treatment compared to the MM_1_ treatment, and the opposite trend was observed for the other three indices.

**Figure 4:**
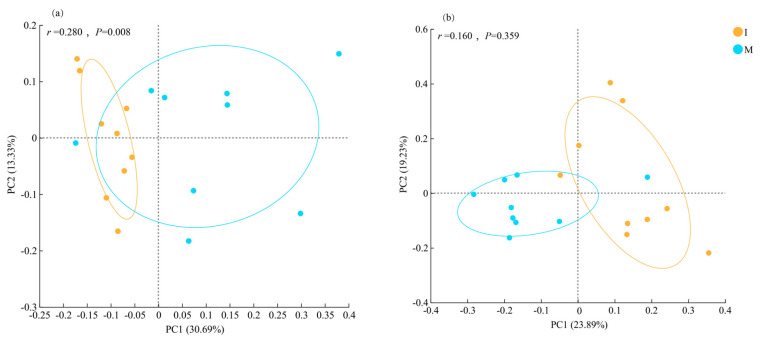
Beta-diversity of soil (**a**) bacterial and (**b**) fungal communities (operational taxonomic unit level) under monocropping (M) and cover cropping (I) revealed by principal co-ordinates analysis based on Bray–Curtis dissimilarity.

PCoA based on Bray–Curtis dissimilarity was conducted to analyze the differences in soil bacterial and fungal community compositions under various cover cropping systems. In the bacterial communities, the percent variance explained by the first (PC1) and second (PC2) axes was 30.69% and 13.33%, respectively. There was a clear separation of samples by treatment, indicating significant difference in bacterial community composition (PERMANOVA, *p* = 0.008; Figure 4a). PC1 and PC2 explained 23.89% and 19.23% of the total variance in fungal communities, respectively, with no significant separation of samples between the two different treatments (PERMANOVA, *p* = 0.0.359; Figure 4b).

### 3.3. Soil Microbial Community Compositions

**Figure 5:**
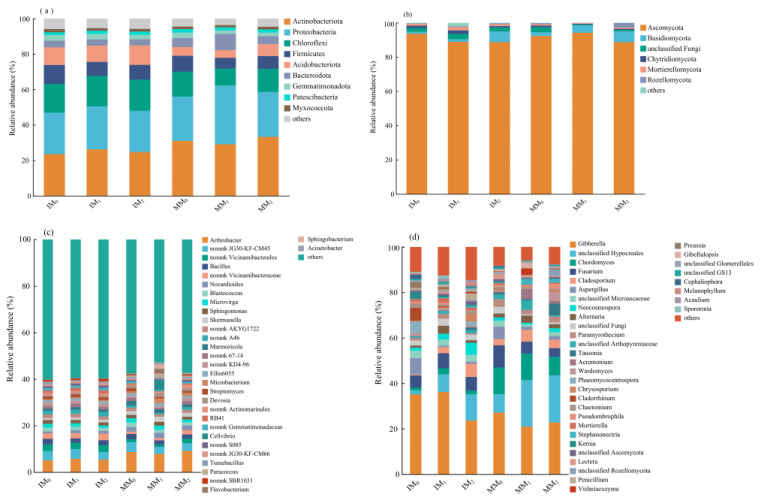
Relative abundance of major phyla (top panel) and genera (bottom panel) in soil (**a**,**c**) bacterial and (**b**,**d**) fungal communities under various treatments.

The community compositions of soil bacteria and fungi were analyzed at two different taxonomic levels (Figure 5). While the dominant phyla and genera remained unchanged, their relative abundances varied across treatments. At the phylum level, the bacterial communities were mainly comprised of Actinobacteriota (23.5–33.3%), Proteobacteria (23.3–33.2%), and Chloroflexi (9.50–17.58%; Figure 5a). Other bacterial phyla included Firmicutes (6.1–10.7%), Acidobacteriota (4.4–11.0%), Bacteroidota (3.4–9.1%), Gemmatimonadota (2.0–3.3%), Myxococcota (1.5–2.0%), and Patescibacteria (1.2–1.8%). In total, these nine phyla accounted for ~96% (or higher) of the total bacterial community, and Actinobacteriota was the dominant group across all treatments. Both Actinobacteriota and Proteobacteria decreased in relative abundance under cover cropping compared to monocropping. In contrast, Chloroflexi, Firmicutes, and Acidobacteriota all increased in response to cover cropping. Irrespective of the cropping system, manure application at medium and high levels led to a decrease in Firmicutes, whereas a high level of manure application led to increase in the relative abundance of Acidobacteriota. The top 30 most abundant bacterial genera accounted for 38.6–47.3% of the total bacterial sequences (Figure 5c). The major genera across all treatments included *Arthrobacter* (5.0–9.1%), *Bacillus* (1.7–2.4%), *Nocardioides* (0.8–2.3%), *Blastococcus* (1.2–2.0%), *Microvirga* (1.0–1.9%), and *Sphingomonas* (1.2–1.6%). Compared to monocropping, cover cropping mainly reduced the relative abundances of *Arthrobacter* and *Nocardioides*, with other genera accounting for higher proportions. A moderate level of manure application resulted in a decrease in the relative abundance of *Arthrobacter* under monocropping (MM_1_), in contrast to the pattern observed under cover cropping (IM_1_). Interestingly, *Bacillus* decreased in relative abundance under manure application in both cropping systems.

In the fungal communities, Ascomycota was the dominant phylum (88.7–94.2%) across all treatments, followed by Basidiomycota (1.2–6.3%; Figure 5b). The cumulative relative abundance of these two dominant fungal phyla ranged from 90.4 to 98.4%. Under cover cropping, Ascomycota and Basidiomycota decreased in relative abundance, whereas other phyla increased, as compared to those of the monocropping system. Notably, the relative abundance of Basidiomycota increased with the increasing level of manure application under different cropping systems. The top 30 most abundant genera accounted for 77.9–93.2% of the total fungal sequences (Figure 5d). The major genera included *Gibberella* (20.9–36.2%), *Chordomyces* (1.2–11.8%), *Fusarium* (3.8–9.8%), *Cladosporium* (0.4–5.8%), *Aspergillus* (0.7–7.1%), *Neocosmospora* (0.8–5.3%), *Alternaria* (0.9–3.5%), and *Penicillium* (0.64–2.72%). *Chordomyces*, *Fusarium*, and *Cladosporium* all decreased in relative abundance in response to cover cropping, accompanied by the increase in other genera. Under different levels of manure application, the relative abundances of *Gibberella* and *Fusarium* decreased, in contrast to the upward trends of other genera.

### 3.4. Soil Microbial Co-Occurrence Patterns

**Figure 6:**
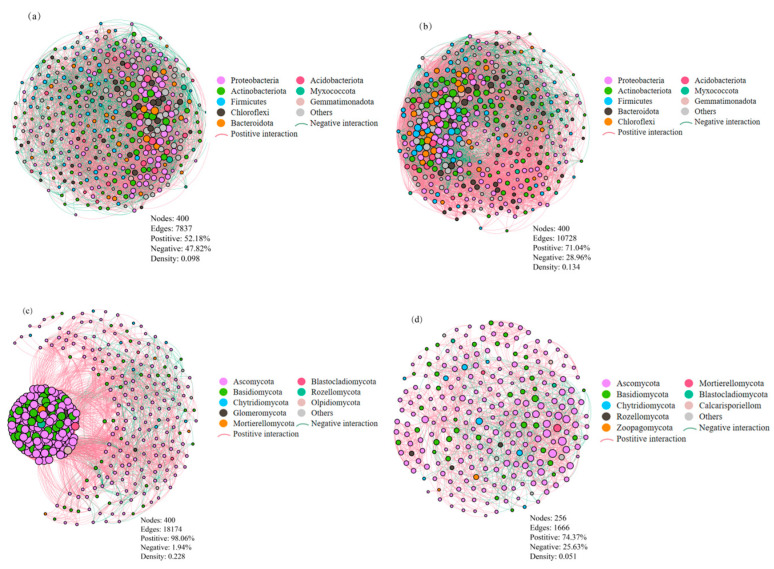
Co-occurrence networks of soil bacterial (top panel) and fungal (bottom panel) taxa under cover cropping (**a**,**c**) and monocropping (**b**,**d**) systems. Each node represents a phylum with an average relative abundance > 0.5%. The size of nodes is proportional to the number of connected edges, and the width of edges is proportional to Spearman’s correlation coefficient.

The co-occurrence networks for soil microbial taxa were constructed for different cropping systems (Figure 6). Each bacterial network contained 400 nodes, with 7837 edges under cover cropping (Figure 6a) and 10,728 edges under monocropping (Figure 6b). There were 4089 positive and 3748 negative correlations in the network of the cover cropping system, with 7621 positive and 3107 negative correlations in the network of the monocropping system. Cover cropping resulted in a lower ratio of positive to negative correlations in the bacterial network (P/N = 1.09) compared to monocropping (P/N = 2.45). This indicates that cover cropping suppressed positive co-occurrence patterns among the soil bacterial communities. Cover cropping additionally diminished bacterial network connectivity, based on a lower graph density of 0.098 compared to that of the monocropping system (0.134). The bacterial network under cover cropping contained six highly connected taxa (connectivity ≥ 4000), i.e., *Blastococcus*, *Arthrobacter*, *Microvirga*, *Vicinamibacterales*, *Vicinamibacteraceae*, and *Sphingomonas*. In the bacterial network under monocropping, 10 highly connected taxa (connectivity ≥ 4000) were found, i.e., *Nocardioides*, *Microvirga*, *Blastococcus*, *Marmoricola*, *Skermanella*, *Arthrobacter*, *Microbacterium*, *Cellvibrio*, *Sphingomonas*, and *Bacillus*.

Fungal network under the cover cropping system consisted of 400 nodes and 18,174 edges, much more than those under monocropping (256 nodes and 1666 edges; Figure 6c,d). In both cases, positive correlations were predominant in the fungal network, with 17,821 positive and 353 negative correlations under cover cropping, and 1239 positive and 427 negative correlations under monocropping. Cover cropping stimulated positive co-occurrence patterns and enhanced network connectivity among fungal taxa. This was indicated by a higher ratio of positive to negative correlations (P/N = 50.5) and graph density (0.228) compared to those of the monocropping system (P/N = 2.9 and graph density = 0.051). Under cover cropping, the fungal network included 16 highly connected taxa (connectivity ≥ 4000), i.e., *Aspergillus*, *Alternaria*, *Paramyrothecium*, *Wardomyces*, *Phaeomycocentrospora*, *Chordomyces*, *Neocosmospora*, *Cladorrhinum*, *Cladosporium*, *Penicillium*, *Fusarium*, *Melanophyllum*, *Kernia*, *Chaetomium*, *Gibberella*, and *Mortierella*. Under monocropping, there were also 16 highly connected taxa in the fungal network (connectivity ≥ 4000), i.e., *Aspergillus*, *Pseudombrophila*, *Parauyrothecium*, *Cladosporium*, *Chordomyces*, *Vishniacozyma*, *Fusarium*, *Lectera*, *Neocosmospora*, *Stephanonectria*, *Gibellulopsis*, *Alternaria*, *Acremonium*, *Gibberella*, *Wardomyces*, and *Tausonia*.

### 3.5. Treatment Effects on Fruit Yield, Soil Properties, and Microbial Diversity

**Figure 7:**
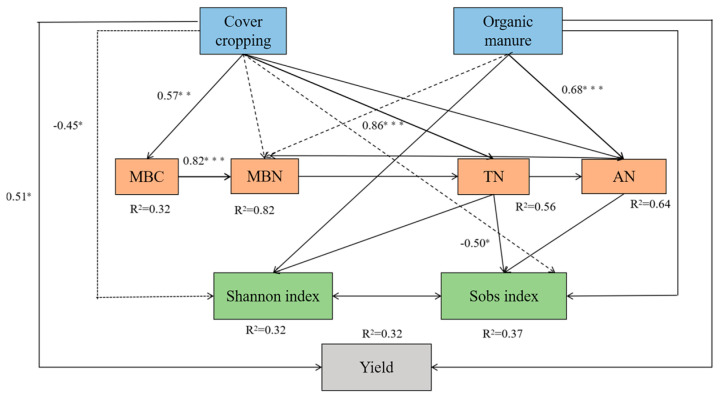
Structural equation modeling reveals the influence of cover cropping and manure application on soil properties, microbial diversity, and *L*. *barbarum* yield. MBC and MBN represent microbial biomass carbon and nitrogen, respectively; TN and AN represent total and available nitrogen, respectively. Positive and negative correlations are represented by solid and dotted arrows, respectively. Significant standardized path coefficients are shown on the arrows. * *p* < 0.05, ** *p* < 0.01, and *** *p* < 0.001.

To decipher the influence of agricultural management practices on soil properties, microbial diversity, and fruit yield, structural equation modeling was performed. The model fit was satisfactory based on the difference divided by degrees of freedom (CMIN/DF = 0.747), goodness-of-fit index (GFI = 0.908), comparative fit index (CFI = 1.000), and root mean square error of approximation (RMSEA = 0.000). While having a direct effect on fruit yield, cover cropping significantly affected the Shannon index and Sobs index through indirect effects on MBC, MBN, TN, and AN. The application of organic manure indirectly affected soil microbial richness (Sobs) by influencing MBN, TN, and AN contents (Figure 7).

## 4. Discussion

### 4.1. Cover Cropping with Manure Alters Soil Microbial Community Structure and Network Complexity

Previous research indicates that planting cover crops in orchards can ameliorate the soil environment by improving microbiota structure and biomass, which in turn enhances soil biological activity. This modern agricultural management practice holds significant importance for maintaining soil health [28]. Generally, a higher microbial diversity supports greater structural complexity and functional stability of soil microbiota [29]. The changes in soil microbiota are driven by a broad range of factors, including soil type, pH, nutrient availability, land management, and crop species [30]. In particular, various cropping systems exhibit distinct effects on soil microbiota, and changes in agricultural management practices alter the community structure of soil bacteria and fungi [31]. Here, our alpha-diversity analysis revealed that planting *R. sativus* as a cover crop increased both the community diversity and richness of soil bacteria in the *L. barbarum* field (Figure 3). This mirrors the results of a meta-analysis conducted by Muhammad et al. [31]. In the presence of cover crops, the application of organic manure, which is rich in humus, can increase soil nutrient contents. As such, organic manure provides additional substrates for soil microbes, potentially enhancing their activities and abundance [32]. Additionally, the C/N ratio of organic manure affects MBC and MBN contents in the soil. When the C/N ratio of organic manure is around 15:1, its N activity is relatively high. However, applying organic manure with an excessively high C/N ratio can considerably reduce the activity of soil N, leading to insufficient N supply. This is detrimental to the growth of soil microbes and consequently impacts microbial diversity [33].

Cover cropping changed soil bacterial community composition in the *L. barbarum* field, as revealed by PCoA analysis (Figure 4). He et al. [34] also observed that applying kudzu vines as green manure improved soil bacterial community structure in young rubber plantations. The phylum Actinobacteriota has been found to be widely distributed in terrestrial ecosystems, particularly in arid soils [35]. We found that the relative abundance of Actinobacteriota decreased in cover-cropped soils (Figure 5a), likely due to the competition with other mesophylic taxa. This pattern might be attributed to increased soil moisture content under cover cropping, as this practice could suppress soil water evaporation and enhance water retention capacity [35]. Additionally, Actinobacteriota produce secondary metabolites, such as phenazines compounds, which allow them to competitive with other microbes [36]. The phylum Proteobacteria contains a large number of oligotrophic functional taxa, with a significant portion of these taxa possessing nitrogen-fixing capabilities. This may be an important reason for the lack of significant differences in the abundance of Proteobacteria in soils under various long-term management practices [37]. Cover cropping led to the loss of *Arthrobacter* and *Nocardioides* in the soil. *Arthrobacter* has nutritional multifunctionality and can degrade environmental pollutants, particularly atrazine in soil [38]. *Nocardioides* spp. are aerobic, acid-nonresistant, mesothermophilic, and neutrophilic bacteria [39].

The dominant phyla in soil fungal communities were Ascomycota and Basidiomycota (Figure 5b). As saprotrophic fungi, these phyla play a crucial role in the decomposition of complex compounds, particularly the break-down of plant residues and degradation of straw remnants [40]. We found that cover cropping increased the relative abundance of Ascomycota while reducing the abundance of Basidiomycota in the soil samples. The possible reason is that cover crops provide additional organic C to the soil through root exudates and residue decomposition. Ascomycota can utilize complex C sources, allowing it to dominate the decomposition and transformation of soil organic matter [41]. The abundance of Ascomycota could lead to a loss of Basidiomycota due to their potentially competitive relationship for nutrients [42]. With regard to the major genera in soil fungal communities (Figure 5d), *Cladosporium* is known to exhibit antimicrobial activity and inhibitory effect against pathogenic fungi, such as *Colletotrichum collosporum*, *Sclerotinia sclerotiorum*, and *Fusarium solani* [43]. *Penicillium* is a group of plant-growth-promoting fungi with phosphate-solubilizing activity, and some species of this genus can induce crop resistance to pathogens by activating multiple defense signaling pathways [44]. *Mortierella* has the ability to improve plant acquisition of bioavailable P and iron in the soil. Some species of *Mortierella* can synthesize plant hormones and protect crops from pathogen attacks [45]. Cover crop cultivation can also effectively reduce pathogenic fungi such as Aspergillus and enhance the disease resistance of crops, which is beneficial for crop growth [46]. Meanwhile, *Gibberella* was enriched in soils under cover cropping, likely due to the increase in soil organic C and AP contents (Table 1). It has been shown that soil organic C and total P contents are key factors influencing the relative abundance of *Gibberella* in forest soils [47].

Co-occurrence network analysis provides a powerful tool that quantifies the relationships between microbial taxa (e.g., nodes) and their strengths. Generally, a high-connectivity network exhibits low stability [48]. Both soil bacterial and fungal networks showed distinct co-occurrence patterns in the *L. barbarum* field under different cropping systems (Figure 6). Cover cropping reduced network connectivity and complexity among the bacterial taxa, whereas the opposite effects were observed for the fungal network, in agreement with the findings of previous studies [31,49]. The planting of cover crops possibly increased soil bacterial diversity and abundance, boosting species competition. The decrease in positive relations resulted in a simpler bacterial network with greater stability. In contrast, soil fungal diversity remained unchanged, and cover cropping facilitated synergistic relationships between fungal taxa, thus increasing network complexity and reducing its stability. Among the highly connected keystone taxa, *Blastococcus*, *Arthrobacter*, and *Microvirga* played a role in maintaining soil bacterial community stability under different cropping systems. *Blastococcus* is a Gram-positive, coccoid, and aerobic genus from the family Geodermatophilaceae [50]. *Microvirga* spp. are beneficial microbes in the soil–plant ecosystem, which can improve soil fertility, stimulate plant growth, and control soil-borne diseases [51]. Additionally, *Aspergillus*, *Alternaria*, and *Wardomyces* contributed to soil fungal community stability under different cropping systems. *Aspergillus* is a diverse genus of ubiquitous fungi that can grow in soil and other settings, some of which are pathogenic to humans and animals [52]. *Alternaria* is a common fungal genus known to cause pre- and postharvest damage to agricultural products, including cereal grains, fruits, and vegetables [53]. The co-occurrence of these bacterial and fungal taxa in the microbial network controls the community stability.

### 4.2. Soil Properties and Fruit Yield Are Improved by Cover Cropping with Manure

Cover cropping in orchards has been found to reduce soil BD, regulate soil temperature, and stimulate soil microbial diversity and enzyme activities [54]. This practice additionally promotes soil nutrient availability and improves nitrogen use efficiency [8]. Similarly, we observed increased soil TN, AN, and AP contents and decreased BD in the *L. barbarum* field planted with *R. sativus* as compared to those of the monocropping system (Table 1). Cover cropping resulted in improved P and K availability in the soil, most likely due to the interaction between *R. sativus* roots and soil nutrients. Root exudates could promote the conversion of insoluble P and K in the soil into bioavailable forms that can be readily absorbed and used by crop plants [22,55].

Weil et al. [56] found that cover cropping with *R. sativus* reduced the loss of nitrogen by leaching from the soil in the Mid-Atlantic. We observed a similar effect in the *L. barbarum*/*R. sativus* planting system, as indicated by increase in soil TN and AN contents (Table 1). *R. sativus* was planted in the beginning of autumn, when *L. barbarum* gradually stopped growing or absorbing soil nutrients. *R. sativus* could capture soil nitrogen through well-developed roots before death in winter, and then release the accumulated nitrogen after decomposition in the next spring. This process would prevent nitrogen leaching and thereby increase soil nitrogen content, providing a rich nitrogen source for the primary crop. Furthermore, the slow release of nitrogen from organic amendment can enable the soil to supply nitrogen over a long period. In particular, crop plants may acquire sufficient nitrogen in the later growth stages. Additionally, manure application can increase the occurrence and activity of soil microbes and allow more inorganic nitrogen to be deposited in the soil through microbial immobilization, which ultimately improves nitrogen use efficiency [57].

Cover crops, as protective crops for the soil, possess root systems with strong penetrating abilities that can improve soil structure and alleviate soil compaction, thereby enhancing the yield of subsequent crops [58]. Various cover crops have different effects on the soil environment and crop yield [59]. Cruciferous cover crops can activate insoluble P in the soil and improve P use efficiency [60], which in turn enhances soil microbial diversity, inhibits soil-borne pests and diseases, and improves soil fertility. Cover cropping with *R. sativus* under the medium level of organic manure markedly increased the fruit yield of *L. barbarum* (Figure 2). Microbial community variations could contribute to soil fertility and nutrient uptake by *L. barbarum*, thus improving its fruit yield. *R. sativus* roots have a strong capacity to penetrate the soil, which is known as biological drilling [5]. This process could facilitate root growth of *L. barbarum* toward the deep soil, thereby promoting soil nutrient absorption and consequently increasing fruit yield.

Complete incorporation of cover crops into the orchard provides a significant component of soil nutrient input. This practice can increase soil organic C content during the growing season. Other benefits include the reduction in nutrient leaching, suppression of weed growth, and control of pests and diseases. All these changes are expected to improve soil quality and increase soil nutrient supply, thereby enhancing the yield and quality of the main crops [61]. The performance of cover crops is the result of multiple factors working together, and the species of cover crops varies across different regions of China. While this study obtained empirical evidence for the use of *R. sativus* cover crops, caution must be taken when using other cover crops with manure for *L. barbarum* production.

## 5. Conclusions

Compared to conventional monocropping of *L. barbarum*, the use of *R. sativus* cover crops altered the structure of soil microbial communities (especially bacteria). While maintaining the same dominant taxa of bacteria and fungi, cover cropping increased the relative abundance of other taxa, thereby enhancing microbial diversity and richness in the soil environment. This reshaping of soil microbial communities promoted nutrient supply for *L. barbarum* plants, ultimately improving fruit yield. The findings of this study can provide guidance for large-scale application of the *L. barbarum/R. sativus* planting system as a scientific soil management model in northwestern China.

## Figures and Tables

**Table 1:** Soil physicochemical properties in various cropping systems under different levels of manure application.

Treatment	pH	BD (g·cm^−3^)	EC (µs·cm^−1^)	TN (g·kg^−1^)	AN (mg·kg^−1^)	AP (mg·kg^−1^)	**AK (mg·kg^−1^)**	**MBC (mg·kg^−1^)**	**MBN (mg·kg^−1^)**
MM_0_	8.97 ± 0.28 a	1.45 ± 0.01 a	109.37 ± 12.81 c	0.76 ± 0.24 b	19.71 ± 7.87 c	15.41 ± 9.74 b	52.65 ± 25.71 b	71.62 ± 16.44 c	13.22 ± 4.84 b
MM_1_	8.85 ± 0.15 a	1.45 ± 0.01 a	133.33 ± 16.98 bc	0.71 ± 0.25 b	35.2 ± 5.74 ab	17.05 ± 4.92 b	38.89 ± 11.42 b	106.27 ± 7.93 c	24.84 ± 10.30 b
MM_2_	8.93 ± 0.34 a	1.45 ± 0.02 a	155.6 ± 30.32 b	0.88 ± 0.44 ab	35.2 ± 12.03 ab	15.84 ± 7.12 b	78.04 ± 35.74 ab	83.57 ± 9.32 c	16.14 ± 5.77 b
IM_0_	9.01 ± 0.02 a	1.42 ± 0.02 ab	116.8 ± 4.79 c	1.34 ± 0.12 ab	27.21 ± 2.71 bc	35.96 ± 10.51 ab	51.00 ± 0.72 b	72.16 ± 17.47 c	24.90 ± 2.71 b
IM_1_	8.97 ± 0.17 a	1.40 ± 0.03 b	136.23 ± 22.61 bc	1.51 ± 0.55 a	40.69 ± 5.28 ab	31.39 ± 15.55 ab	74.59 ± 18.55 ab	225.80 ± 18.33 a	89.65 ± 10.18 a
IM_2_	8.85 ± 0.13 a	1.45 ± 0.01 a	191.07 ± 10.57 a	1.33 ± 0.28 ab	49.50 ± 8.37 a	47.63 ± 15.77 a	114.47 ± 31.43 a	170.09 ± 11.45 b	39.43 ± 10.09 b
A	ns	*	ns	***	ns	***	ns	*	**
B	ns	ns	***	ns	**	ns	*	*	*
A × B	ns	ns	**	ns	**	*	*	***	***

pH: pondus hydrogenii; BD: bulk density; EC: electrical conductivity; TN: total nitrogen; AN: available nitrogen; AP: available phosphorus; AK: available potassium; MBC: microbial biomass carbon; and MBN: microbial biomass nitrogen. MM_0_, MM_1_, and MM_2_ are the monocropping treatments and IM_0_, IM_1_, and IM_2_ are the cover cropping treatments, with zero, medium, and high levels of organic manure, respectively. A: Cropping system; B: Manure application; A × B: Cropping system × Manure application (ns: not significant, * *p* < 0.05, ** *p* < 0.01, and *** *p* < 0.001). Data represent the means (*n* = 3). In each column, different lowercase letters indicate significant differences among the treatments (Duncan’s multiple range test: *p* < 0.05).

## Data Availability

The original contributions presented in the study are included in the article/Supplementary Material, further inquiries can be directed to the corresponding authors.

## Supplementary Materials

The following supporting information can be downloaded at: https://www.mdpi.com/article/10.3390/microorganisms13030696/s1. Table S1: Sequencing results of soil bacteria and fungi under cover cropping and manure treatments; Figure S1: Rarefaction curves of (a) bacterial and (b) fungal communities in soil samples of different treatment groups (defined in Table S1 footnote).

## References

[1] Zhu Y., Chen B., Fu W. (2022). Research Frontiers in Soil Ecology. Sci. Technol. Rev..

[2] Huang Y., Luo F., Gong X., Wang Y., Li L., Liu D., Yao Y. (2023). Effects of Organic Fertilizers on Soil Microbial Community Characteristics: Research Progress. Chin. Agric. Sci. Bull..

[3] Zhao J., Ma J., Yang Y., Yu H., Zhang S., Chen F. (2021). Response of Soil Microbial Community to Vegetation Reconstruction Modes in Mining Areas of the Loess Plateau, China. Front. Microbiol..

[4] Hartmann M., Six J. (2023). Soil Structure and Microbiome Functions in Agroecosystems. Nat. Rev. Earth Environ..

[5] Wang F., Li W.H., Chen H.N., Weil R., Zhu L.Z., Nan X.X. (2023). Forage Radish Cover Crops Improve Soil Quality and Fruit Yield of *Lycium barbarum* L. in An Arid Area of Northwest China. Agronomy.

[6] Ma J.J., Yao H., Liu H., Tian M.R. (2023). Evolution Characteristics of Soil Nutrients and Microorganisms During Alfalfa Restoration of Mining Area in Yanshan Mountain. J. Environ. Eng. Technol..

[7] Li Y.X., Wang S.D., Ke Y., Luo J.H., Chen X.Q., Zhang X.J. (2016). Characteristics of Soil Nutrients and Present Situation of Fertilization in the Major Wolfberry Producing Areas of Ningxia. Agric. Res. Arid Areas.

[8] Dikgwatlhe S., Kong F., Chen Z., Lal R., Chen F. (2014). Tillage and Residue Management effects on Temporal Changes in Soil Organic Carbon and Fractions of a Silty Loam Soil in the North China Plain. Soil Use Manag..

[9] Rusu T. (2014). Energy Efficiency and Soil Conservation in Conventional, Minimum Tillage and No-tillage. Int. Soil Water Conserv. Res..

[10] Zhang Z.B., Peng X.H. (2021). Bio-tillage: A New Perspective for Sustainable Agriculture. Soil Tillage Res..

[11] Ding T.T., Duan T.Y. (2021). Research Progress on the Influence of Orchard Green Manure on Fruit Tree Soil-microbe System. J. Fruit Sci..

[12] Wang Y., Liu L., Tian Y., Wu X., Yang J., Luo Y., Li H., Awasthi M., Zhao Z. (2020). Temporal and Spatial Variation of Soil Microorganisms and Nutrient Under White Clover cover. Soil Tillage Res..

[13] Jin X., Wang Y., Liu C., Chen X., Long M., He S. (2022). Effects on Soil Nutrients and Bacterial Communities of Different Cover Crops in an Organic Kiwifruit Orchard in the Guanzhong Region of China. Acta Prataculturae Sin..

[14] Zhong Z., Huang X., Feng D., Xing S., Weng B. (2018). Long-term Effects of Legume Mulching on Soil Chemical Properties and Bacterial Community Composition and Structure. Agric. Ecosyst. Environ..

[15] Sun R.B., Chen Y., Han W.X., Dong W.X., Zhang Y.M., Hu C.S., Liu B.B., Wang F.H. (2020). Different Contribution of Species Sorting and Exogenous Species Immigration from Manure to Soil Fungal Diversity and Community Assemblage under long-term Fertilization. Soil Biol. Biochem..

[16] Sedghi N., Weil R. (2022). Fall Cover Crop Nitrogen Uptake Drives Reductions in Winter-spring Leaching. J. Environ. Qual..

[17] Wang F., Weil R., Nan X. (2017). Total and Permanganate-oxidizable Organic Carbon in the Corn Rooting Zone of US Coastal Plain Soils as Affected by Forage Radish Cover Crops and N fertilizer. Soil Tillage Res..

[18] Peng T., Ma S., Ma C., Song Y., Gao N., Li K., Zhang C., Li J., Na X., Wang L. (2023). Effects of Long-term Monocropping on Soil Microbial Metabolic Activity and Diversity in Topsoil and Subsoil Horizons of *Lycium barbarum* Fields. Acta Prataculturae Sin..

[19] Bao S. (2005). Soil Analysis Manual.

[20] Xu N., Tan G., Wang H., Gai X. (2016). Effect of Biochar Additions to Soil on Nitrogen Leaching, Microbial Biomass and Bacterial Community Structure. Eur. J. Soil Biol..

[21] Adams R., Miletto M., Taylor J., Bruns T. (2013). Dispersal in Microbes: Fungi in Indoor Air are Dominated by Outdoor Air and Show Dispersal Limitation at Short Distances. ISME J..

[22] Chen G., Weil R.R. (2011). Root Growth and Yield of Maize as Affected by Soil Compaction and Cover crops. Soil Tillage Res..

[23] Magoč T., Salzberg S.L. (2011). FLASH: Fast Length Adjustment of Short Reads to Improve Genome Assemblies. Bioinformatics.

[24] Edgar R.C. (2013). UPARSE: Highly Accurate OTU Sequences from Microbial Amplicon Reads. Nat. Methods.

[25] Stackebrandt E., Goebel B.M. (1994). Taxonomic Note: A Place for DNA-DNA Reassociation and 16S rRNA Sequence Analysis in the Present Species Definition in Bacteriology. Int. J. Syst. Bacteriol..

[26] Wang Q., Garrity G.M., Tiedje J.M., Cole J.R. (2007). Naive Bayesian Classifier for Rapid Assignment of rRNA Sequences Into the New Bacterial Taxonomy. Appl. Environ. Microbiol..

[27] Kou R., Feng G.Y., Li C., Dong H.Y., Fan X.J. (2023). The Composition, Interaction Network, and Source of Microbia Community in Shanxi Mature Vinegar Produced by Liquid-solid Fermentation. Microbiol. China.

[28] Blanco C.H., Shaver T.M., Lindquis J., Shapiro C., Elmore R., Francis C., Hergert G. (2015). Cover Crops and Ecosystem Services: Insights from Studies in Temperate Soils. Agron. J..

[29] Rashid M.I., Mujawar L.H., Almeelbi T., Shahzad T., Almeelbi T., Ismail I.M.I., Oves M. (2016). Bacteria and Fungi can Contribute to Nutrients Bioavailability and Aggregate Formation in Degraded Soils. Microbiol. Res..

[30] Romaniuk R., Giuffré L., Costantini A., Nannipieri P. (2011). Assessment of Soil Microbial Diversity Measurements as Indicators of Soil Functioning in Organic and Conventional Horticulture Systems. Ecol. Indic..

[31] Muhammad I., Wang J., Sainju U., Zhang S., Zhao F., Khan A. (2021). Cover Cropping Enhances Soil Microbial Biomass and Affects Microbial Community Structure: A meta-analysis. Geoderma.

[32] Xiong H., Liu Y., Li Y., Zhang Y., Huang X., Yang Y., Zhu H., Jiang T. (2023). Effects of Long-term fertilization Patterns on Bacterial Community Structure and Soil Nutrients in Dryland of Yellow Soil. Chin. J. Appl. Ecol..

[33] Xu Z.S., Liu J.H., Lu X.P., Chen X.J., Zhang B.W., Zhang X.L., Zhu W., Yang Y.M. (2020). The Application of Organic Fertilizer Improves the Activity of the Soil Enzyme, Increases the Number and the Species Variety of Bacteria in Black Soil. Soil Fertiltzer Sci. China.

[34] He J., Zhang H., Luo W., Zhang P., Li W., Luo P. (2021). Soil Bacterial Community Structure Improved in Young Rubber Plantation Covered with Kudzu vines. Soil Fertil. Sci. China.

[35] Liu Z.H., Huang F.Y., Li J.L., Zhang P., Yang B.P., Ding R.X., Nie J.F., Jia Z.K. (2021). Effects of Farmland Mulching Patterns on Soil Microbial Diversity and Community Structure in Dryland. Acta Ecol. Sin..

[36] Yan B.F., Liu N., Liu M.H., Du X.Y., Shang F., Huang Y. (2021). Soil Actinobacteria Tend to Have Neutral Interactions with other Coccurring Microorganisms, Especially under Oligotrophic Conditions. Environ. Microbiol..

[37] Liu J.J., Sui Y.Y., Yu Z.H., Shi Y., Chu H.Y., Jin J., Liu X.B., Wang G.H. (2014). High Throughput Sequencing Analysis of Biogeographical Distribution of Bacterial Communities in the Black Soils of Northeast China. Soil Biol. Biochem..

[38] Zhang X.J., Zhang G.Z., Yang H.T. (2016). Genomics Basis of Arthrobacter Spp Environmental Adaptability—A review. Acta Microbiol. Sin..

[39] Du H.J., Yu L.Y., Zhang Y.Q. (2012). Recent Advance on the Genus Nocardioides—A review. Acta Microbiol. Sin..

[40] Bastida F., Torres I.F., Moreno J.L., Baldrian P., Ondoño S., Ruiz N.A., Hernández T., Richnow H.H., Starke R., García C. (2016). The Active Microbial Diversity Drives Ecosystem Multifunctionality and is Physiologically Related to Carbon Availability in Mediterranean Semi-arid soils. Mol. Ecol..

[41] Kõljalg U., Nilsson R.H., Abarenkov K., Tedersoo L., Taylor A.F., Bahram M., Bates S.T., Bruns T.D., Bengtsson-Palme J., Callaghan T.M. (2013). Towards a Unified Paradigm for Sequence-based Identification of Fungi. Mol. Ecol..

[42] Geng H.T., Wang X.D., Shi S.B., Yi Z.Q., Zhou W.J. (2023). Effects of Combined Application of Fungal Residue and Chemical Fertilizer on Soil Microbial Community Composition and Diversity in Paddy soil. Environ. Sci..

[43] Huang Q.E., Wu T., Zhang K.J., He W.Y., Cao J.Q., Xu J.Y., Qing L.P., Zhu B. (2024). Fungal Community Structure Diversity and Biological Activity of Culturable Endophytic Fungi from Actinidia Eriantha. Chin. Tradit. Herb. Drugs.

[44] Hossain M., Sultana F., Kubota M., Koyama H., Hyakumachi M. (2007). The Plant Growth-Promoting Fungus Penicillium Simplicissimum GP17-2 Induces Resistance in Arabidopsis Thaliana by Activation of Multiple Defense Signals. Plant Cell Physiol..

[45] Ozimek E., Hanaka A. (2021). *Mortierella* Species as the Plant Growth-Promoting Fungi Present in the Agricultural Soils. Agriculture.

[46] Sharma S.B., Sayyed R.Z., Trivedi M.H., Gobi T.A. (2013). Phosphate Solubilizing Microbes: Sustainable Approach for Managing Phosphorus Deficiency in Agricultural Soils. SpringerPlus.

[47] Wang S.H., Chang S.L., Li X., Zhang Y.T. (2021). Soil Fungal Diversity and its Community Structure in Tianshan Forest. Acta Ecol. Sin..

[48] Jiang P., Wang Y.Z., Zhang Y.P., Fei J.C., Rong X.M., Peng J.W., Yin L.C., Zhou X., Luo G.W. (2024). Enhanced Productivity of Maize Through Intercropping is Associated with Community Composition, Core Species, and Network Complexity of Abundant Microbiota in Rhizosphere Soil. Geoderma.

[49] Cazzaniga S.G., Braat L., Elsen S.V.D., Lombaers C., Visser J., Obinu L., Macia-Vicente J.G., Postma J., Mommer L., Helder J. (2023). Pinpointing the Distinctive Impacts of Ten cover Crop Species on the Resident and Active Fractions of the Soil Microbiome. Appl. Soil Ecol..

[50] Normand P., Daffonchio D., Gtari M., Rosenberg E., DeLong E.F., Lory S., Stackebrandt E., Thompson F. (2014). The Family Geodermatophilaceae. The Prokaryotes.

[51] Yang J., Wang Y., Cui X.Y., Xue K., Zhang Y., Yu Z. (2019). Habitat Filtering Shapes the Differential Structure of Microbial Communities in the Xilingol Grassland. Sci. Rep..

[52] Gómez S., Fernández F.J., Vega M.C. (2016). Chapter 4–Heterologous Expression of Proteins in *Aspergillus*. New and Future Developments in Microbial Biotechnology and Bioengineering.

[53] Dall’Asta C., Cirlini M., Falavigna C. (2014). Chapter Three–Mycotoxins from Alternaria: Toxicological Implications. Advances in Molecular Toxicology.

[54] Zhang J.H., Zhang Y.X., Hou S.S., Li H.B., Zhang R.F., Wang H., Wang X.X. (2023). Research Progress on Benefits and Rational Selection of Cover Crops. Trans. Chin. Soc. Agric. Eng..

[55] Yang L., Bai J., Liu J., Zeng N., Cao W. (2018). Green Manuring Effect on Changes of Soil nitrogen Fractions, Maize Growth and Nutrient Uptake. Agronomy.

[56] Weil R., White C., Lawley Y. (2009). Forage radish: New Multi-Purpose Cover Crop for the Mid-Atlantic. Fact Sheet.

[57] Wei Y., Bo Q., Tang A., Gao J., Ma T., Wei X., Zhang F., Zhou X., Yue S., Li S. (2023). Effects of Long-term Film Mulching and Application of Prganic Fertilizer on Yield and Quality of Spring Maize on the Loess Plateau. Sci. Agric. Sin..

[58] Welch R.Y., Behnke G.D., Davis A.S., Masiunas J. (2016). Using Cover Crops in Headlands of Organic Grain Farms: Effects on Soil Properties, Weeds and Crop Yields. Agric. Ecosyst. Environ..

[59] Connell R., Zeglin L., Blair J. (2021). Plant Legacies and Soil Microbial Community Dynamics Control Soil Respiration. Soil Biol. Biochem..

[60] Gao S., Gao J., Cao W., Zou C., Huang J., Bai J., Dou F. (2018). Effects of Long-term Green Manure Application on the Content and Structure of Dissolved Organic Matter in Red Paddy Soil. J. Integr. Agric..

[61] Du W., Wang Z.Q., He W.X., Gao Y.J., Cao W.D. (2017). Effects of Leguminous Green Manure on Soil Nutrients and Their Ecological Stoichiometry Characteristics in Weibei Rainfed Highland. Acta Pedol. Sin..

