# Supplementary material for: Integrating Cover Crops and Manure to Boost Goji Berry Yield: Responses of Soil Physicochemical Properties and Microbial Communities
Source: Microorganisms. 2025 Mar 20;13(3):696. doi: 10.3390/microorganisms13030696 (PMC11944604; doi:10.3390/microorganisms13030696)
Supplement: Supplementary file 1 [file microorganisms-13-00696-s001.zip › microorganisms-3492745-supplementary.pdf]

Supplementary material for

Integrating Cover Crops and Manure to Boost Goji Berry Yield: Responses of Soil Physicochemical Properties and Microbial Communities

Table S1. Sequencing results of soil bacteria and fungi under cover cropping and manure treatments.

Treatment	Bacteria			Fungi		
	Optimized tag	Effective tag	Proportion	Optimized tag	Effective tag	Proportion
MM_0	53972	22821	42.67	59241	36350	61.36
MM_1	53484	22695	42.43	72137	42311	58.65
MM_2	57962	29392	50.71	72225	44770	61.99
IM_0	47459	26887	56.65	71391	27001	37.82
IM_1	50895	26858	52.77	73308	38196	52.10
IM_2	52223	27779	53.19	72096	32344	44.86
Total	315995	156432		420398	220972	

Abbreviations: MM_0, MM_1, and MM_2 are the monocropping treatments, and IM_0, IM_1, and IM_2 are the intercropping treatments, with zero, medium, and high level of organic manure, respectively.

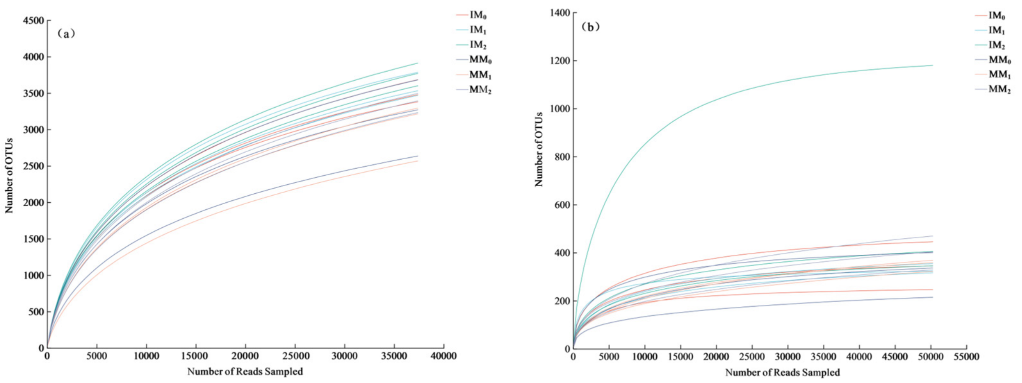

Figure S1. Rarefaction curves of (a) bacterial and (b) fungal communities in soil samples of different treatment groups (defined in Table S1 footnote).
